# Pregnancy-related myocardial infarction

**DOI:** 10.1007/s12471-017-0989-9

**Published:** 2017-04-19

**Authors:** H. Lameijer, M. C. Lont, H. Buter, A. J. van Boven, P. W. Boonstra, P. G. Pieper

**Affiliations:** 1 Department of Cardiology, University Medical Center Groningen, University of Groningen, Groningen, The Netherlands; 2 Department of Emergency Medicine, University of Groningen, UMCG Groningen, Groningen, The Netherlands; 3 Department of Intensive Care, Medical centre Leeuwarden, Leeuwarden, The Netherlands; 4 Department of Cardiology and Cardiothoracic Surgery, Medical Centre Leeuwarden, Leeuwarden, The Netherlands

**Keywords:** Pregnancy, Myocardial infarction, Ischaemic heart disease, Gender

## Abstract

**Introduction:**

The risk of acute myocardial infarction in young women is low, but increases during pregnancy due to the physiological changes in pregnancy, including hypercoagulability. Ischaemic heart disease during pregnancy is not only associated with increased maternal morbidity and mortality, but also with high neonatal complications. Advancing maternal age and other risk factors for cardiovascular diseases may further increase the risk of ischaemic heart disease in young women.

**Methods:**

We searched the coronary angiography database of a Dutch teaching hospital to identify women with acute myocardial infarction who presented during pregnancy or postpartum between 2011 and 2013.

**Results:**

We found two cases. Both women were in their early thirties and both suffered from myocardial infarction in the postpartum period. Acute myocardial infarction was due to coronary stenotic occlusion in one patient and due to coronary artery dissection in the other patient. Coronary artery dissection is a relatively frequent cause of myocardial infarction during pregnancy. Both women were treated by percutaneous coronary intervention and survived.

**Conclusion:**

Physicians should be aware of the increased risk of myocardial infarction when encountering pregnant or postpartum women presenting with chest pain.

## Introduction

Ischaemic heart disease (IHD) and acute myocardial infarction in fertile women are rare [1]. However, pregnancy greatly increases the risk for IHD in these women [2, 3]. This can be explained by the physiological changes in pregnancy, including a hyperdynamic circulation and hypercoagulability. Advancing maternal age and other risk factors for cardiovascular diseases may further increase the risk of IHD in young women [4, 5]. IHD during pregnancy is not only associated with increased maternal morbidity and mortality, but also with high neonatal complications [2, 6, 7]. Information about IHD during pregnancy or the postpartum period is scarcely available and mainly consists of case reports, two studies, and few reviews [2, 6–9]. While the treatment of IHD advances, contemporary cases of pregnancy-related IHD are scarce [7]. We therefore present two recent cases of acute myocardial infarction presenting during pregnancy or in the postpartum period.

## Methods

We searched the coronary angiography database of the Department of Cardiology of the Medical Centre Leeuwarden, a teaching hospital in Leeuwarden, the Netherlands. We selected fertile women (defined as <45 years) who underwent coronary angiography between March 2011 and March 2013. Women who underwent coronary angiography during pregnancy or up to three months postpartum were included. We searched their medical files for proven IHD, coronary artery disease or acute myocardial infarction, based on coronary angiography results during pregnancy and up to three months postpartum.

## Report

Fourteen young, fertile women underwent coronary angiography; two women met our inclusion criteria.

### Case 1

**Fig. 1:**
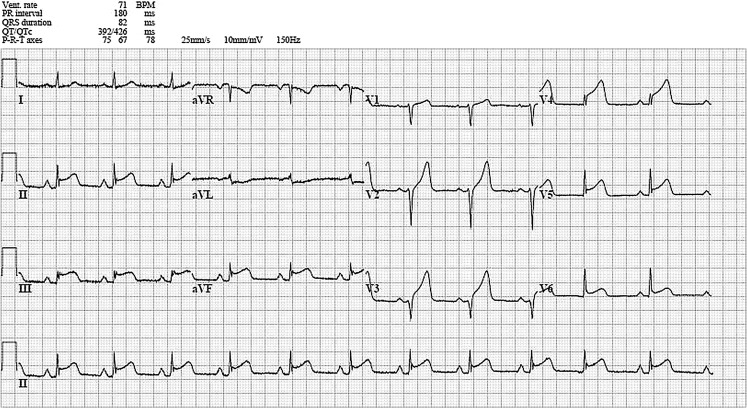
An ECG showing ST segment elevation in leads II, III, aVF and V2–V5 and minimal ST segment depression in aVL, suggesting panischaemia

**Fig. 2:**
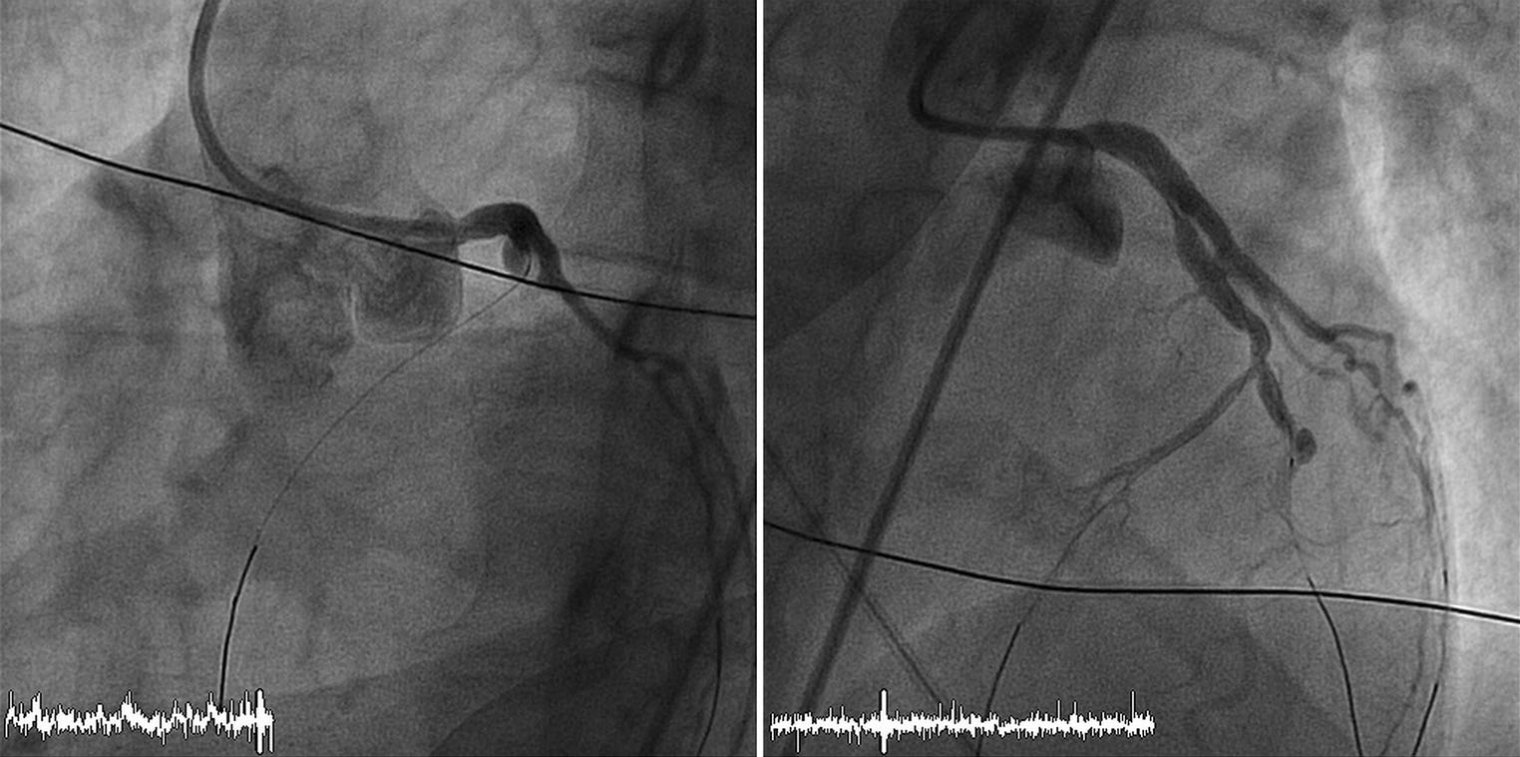
A coronary angiography of patient 1, showing occlusion of the left anterior descending artery distally from the first diagonal artery before and after treatment

A 31-year-old woman, gravida 8 para 4 (G8P4), presented at our cardiology department with chest pain three weeks after delivery of a healthy neonate. She had a history of alcohol and recreational drug abuse (cocaine and amphetamine). Other risk factors for cardiovascular diseases were smoking, hypertension and hypercholesterolaemia. She presented with chest pain, and additional complaints were nausea, vomiting, excessive perspiration and epigastric pain. Physical examination showed a pale woman with clammy skin. She was hypotensive (blood pressure 87/53 mm Hg) with a heart rate of 60 beats per minute. Cardiac auscultation showed no abnormalities. Electrocardiogram (ECG) showed acute ST elevation myocardial infarction (STEMI) (Fig. 1). Echocardiography showed a moderately reduced left ventricular function with akinesia of the septal, anterior and distal inferior wall, without signs of pericardial effusion. A coronary angiography was performed (Fig. 2). She was treated with bare metal stenting. Creatine kinase (CK) levels were elevated to 3760 U/l, CK-MB levels to 217 U/l. A toxicology screening was performed at presentation and she tested negative for cocaine or other recreational drugs. She was discharged after five days; her condition was stable. During follow-up, she was admitted to a cardiac rehabilitation programme and was encouraged to alter her high-risk lifestyle. Echocardiography during follow-up showed an estimated left ventricular ejection fraction of 40–45%.

### Case 2

**Fig. 3:**
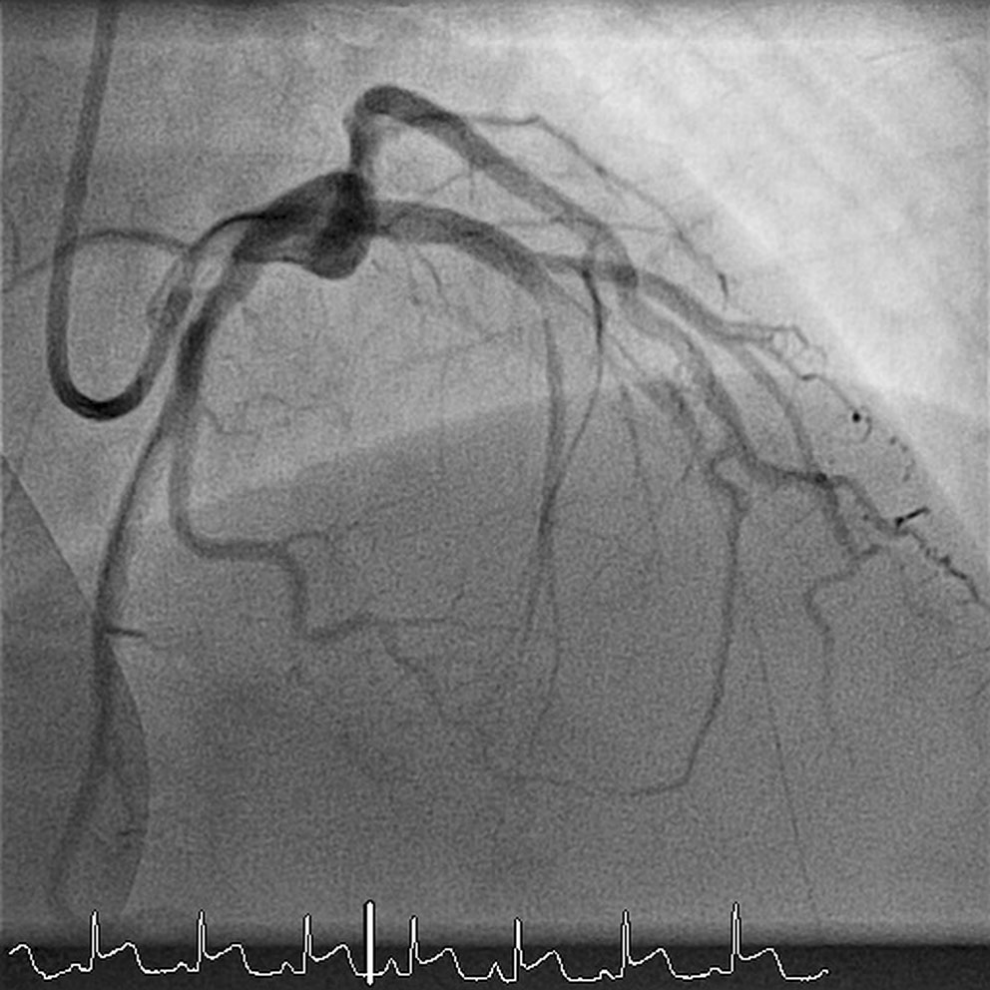
A coronary angiography of patient 2, showing an occlusion of the left main coronary artery and a dissection of the left anterior descending artery and circumflex coronary artery

A 30-year-old woman, G3P3, with a history of migraine headaches for which she incidentally used tramadol and acetaminophen, presented at our emergency department three months postpartum. She complained about chest pain and excessive perspiration. Pain diminished after administration of nitroglycerine sublingually. Physical examination revealed that she was in shock; she had a systolic blood pressure of 90 mm Hg and a regular tachycardia of 120 beats per minute. She also had a pale, cold skin. Auscultation revealed no cardiac murmurs. ECG suggested a STEMI of the anterior wall. Coronary angiography was performed and revealed an occlusion of the left main coronary artery and a dissection of the left anterior descending artery and circumflex coronary artery (Fig. 3). During coronary angiography, external defibrillation was applied twice to treat ventricular fibrillation. Echocardiography after the coronary angiography showed akinesia of the anterior wall and mitral valve regurgitation grade II. Because of her compromised haemodynamic state despite the initiation of inotropics, an intra-aortic balloon pump was inserted. Emergency coronary artery bypass grafting was performed with a left internal mammary artery graft to the left anterior descending artery and a saphenous venous graft to the anterolateral and obtuse marginal branches. A subsequent postoperative cardiogenic shock was treated with the intra-aortic balloon pump for one day and inotropics for two days. Her condition steadily improved and she was discharged from the intensive care unit after five days. At hospital discharge, ten days after admission, echocardiography showed a moderately reduced left ventricular function without valvular regurgitation. At follow-up, three weeks after discharge, she was in stable condition without any signs or symptoms of ischaemia or heart failure.

## Discussion

We identified two cases of pregnancy-related IHD in a teaching hospital over a two-year period. As previously described in this journal, pregnancy-related IHD is rare, with an incidence of 2.8 to 6.2 per 100,000 deliveries described in recent reviews [2, 3, 6]. In this large teaching hospital, only 14 women of fertile age underwent a coronary angiography during the period searched, and two of them (14%) had pregnancy-related IHD. One of our patients had several risk factors for IHD, consistent with the literature where a high prevalence of risk factors is reported in pregnancy-associated IHD, specifically when atherosclerotic disease is present [7]. Our second patient, who had a coronary artery dissection, however, had no risk factors for coronary artery disease, which is again consistent with current literature [7]. Coronary artery dissection, which is rare outside pregnancy, is one of the main aetiologies of acute myocardial infarction during pregnancy or the postpartum period [7].

Both women presented with chest pain in the postpartum period. This is consistent with the literature, where the majority of cases of acute myocardial infarction during pregnancy present with chest pain in the third trimester or the postpartum period and predominantly involve the anterior myocardial wall [2, 3, 6].

Both women were treated successfully for IHD and survived. Myocardial infarction during or shortly after pregnancy is a very high-risk condition with maternal mortality rates ranging from 5.1 to 11% [2, 3, 6]. When a pregnant woman presents with chest pain, the diagnoses that should be considered are pulmonary embolism, aortic dissection and myocardial infarction. ECG and troponin levels should be assessed to diagnose infarction, while echocardiography and computerised tomography are needed to diagnose aortic dissection and pulmonary embolism. Percutaneous coronary intervention is the preferred treatment in women with STEMI or non-STEMI who have risk factors, according to current guidelines [8]. Bare metal stents are preferred over drug-eluting stents in pregnant women, because prolonged dual antiplatelet therapy is preferably avoided [8, 9]. In stable patients with coronary artery dissection a more conservative approach has been advocated, since spontaneous healing often occurs and percutaneous coronary intervention is fraught with technical difficulties and a high failure rate [10]. Medical treatment may include beta blockers and acetylsalicylic acid. Clopidogrel, though safe in animal studies, should be used with caution since experience in humans is limited. ACE-inhibitors and angiotensin receptor blockers are contra-indicated during pregnancy. Vaginal delivery is usually appropriate [11]. Standard IHD risk factor management such as reducing smoking habits, obesity, hypertension and hypercholesterolaemia and treating lipoprotein disorders should be implemented. Additionally, antiphospholipid syndrome as a contributor to myocardial infarction in young women with a history of pregnancy morbidity such as spontaneous abortions, as observed in our first case, should be evaluated [12].

## Conclusion

Physicians should be aware of this increased risk of manifestations of IHD when encountering young pregnant or postpartum women with chest pain.
